# The Effectiveness of Supportive Psychotherapy in Weight Loss in a Group of Young Overweight and Obese Women

**DOI:** 10.3390/nu13020532

**Published:** 2021-02-06

**Authors:** Krzysztof Juchacz, Patrycja Kłos, Violetta Dziedziejko, Rafał W. Wójciak

**Affiliations:** 1 Department of Clinical Psychology, Poznan University of Medical Study, 60-812 Poznań, Poland; juchaczkrzysztof@gmail.com; 2 Department of Biochemistry and Medical Chemistry, Pomeranian Medical University, 70-111 Szczecin, Poland; viola@pum.edu.pl

**Keywords:** women, obesity, slimming, psychotherapy

## Abstract

Overweight and obesity are among the most widespread health problems worldwide. The primary cause of obesity is an inability to control overeating. Therefore, today, obesity needs to be treated more as an eating disorder, i.e., a mental disorder, and thus, it should be approached as such. Taking the above together, this study aimed to assess the impact of supportive psychotherapy on reducing body weight in young overweight and obese women who attempted slimming therapy and, additionally, the possibility of maintaining the weight-loss effect in the long term. Sixty young women aged 20–30 were randomized into three groups that differed in therapeutic management. With the help of an individually selected diet plan, the highest effectiveness in weight loss was demonstrated in people whose weight reduction was supported by goal-oriented psychotherapy. In this group, a sustained effect of slimming and even further weight loss were observed six months following the discontinuation of the therapy. In conclusion, traditional slimming therapies using an individual diet plan and a dietitian’s care are effective; however, supportive psychotherapeutic work provides more beneficial results and maintains the change from a long-term perspective.

## 1. Introduction

Obesity and overweight are increasingly severe problems worldwide, approaching a global epidemic (also called “globesity”) [1]. These problems are being noticed in younger and younger populations in developed and developing countries, even affecting teenagers and children [2,3]. Overweight and obesity, apart from being an aesthetic issue, are significant causes of many comorbidities, such as type 2 diabetes, certain types of cancers (e.g., colorectal and endometrial cancer, or oesophageal adenocarcinoma), hypertension, congestive heart failure, stroke, and hypercholesterolaemia [4,5,6], which, in turn, raise obesity-related medical care costs [7]. In addition to physical consequences, there is also a wide range of mental health conditions connected with obesity—depression, body image disorders, stress, and low self-esteem, to name only a few [8]. Moreover, overweight and obese individuals often experience weight-related discrimination or stigmatization [9]. All in all, both physical and mental overweight-/obesity-associated conditions may significantly reduce the quality of life of persons with increased body weight.

The main reason for excessive body weight is energy consumption from food above the requirements of the body. Accordingly, traditional interventions aimed at fighting overweight/obesity include dietary approaches (mainly based on calorie restriction) combined with increased physical activity and/or pharmacological/surgical treatment [10,11,12,13,14,15,16,17]. Among dietary strategies, a very-low-calorie ketogenic diet seems to be particularly desirable in fighting obesity, as it guarantees rapid weight loss without affecting muscle mass [13]. Nevertheless, a sole dietary intervention, regardless of the type of diet, brings short-term effects, and long-term weight-loss maintenance remains a challenge [18]. Surgical treatment (bariatric surgery) has proved useful in achieving such a long-term effect in morbidly obese patients. Nevertheless, 20–30% of individuals who have undergone bariatric surgery have to face premature weight stabilization or even weight regain if the dietary/lifestyle habits leading to obesity remain unchanged [15].

Apart from overeating, there may be a psychological factor involved in obesity pathogenesis [19,20]. In our modern obesogenic environment, food is no longer just a tool to satisfy hunger. It is also a crucial substitute element in reducing the deprivation of needs other than the need for nutrition, such as, for example, the need for emotional contact. Additionally, for some individuals, food may serve as an instrument for pushing aside unresolved problems or distracting people from their negative emotions. Furthermore, eating disturbances—e.g., compulsive overeating that may lead to excessive energy intake—combine the features of both chemical and process addictions, which are routinely treated with psychotherapy [21]. For this reason, more and more psychologists, psychotherapists, and dieticians believe that overweight and obesity are a consequence of eating disorders and that psychotherapy should be included in the fight against them [15,18,21,22,23,24,25]. Among several psychotherapeutic approaches proposed for the treatment of obesity, cognitive behavioural therapy (CBT) and different forms of behavioural therapy (BT) are the most preferred ones [15,18,26,27,28,29]. CBT has proven its short-term efficacy in treating eating disorders that may be the underlying causes of obesity, such as emotional eating or binge eating disorder [27,30]. However, the results demonstrating its effectiveness in weight reduction and long-term weight-loss maintenance remain inconclusive, mainly because of non-homogeneous therapeutic strategies [15,28]. Additionally, little is known about the efficacy of other psychotherapeutic approaches in reducing/maintaining normal body weight. Accordingly, we aimed to assess the impact of supportive psychotherapy on body weight reduction and long-term weight-loss maintenance in young overweight and obese women undergoing slimming therapy. Our psychotherapeutic strategy of choice was a psychodynamic psychotherapy approach.

## 2. Materials and Methods

### 2.1. Participants

The study’s subjects were 65 young women, volunteers aged 23–25 (mean, 23.6 ± 0.8). After the first part of the experiment, five women withdrew from further work. The remaining women were randomly assigned to four groups of 15 people each. All the persons had body weights greater than recommended, classifying the women as overweight or obese. The subjects were informed of the research’s purpose and procedure and provided written, informed consent to participate in the study. All the study procedures were conducted in accordance with the Declaration of Helsinki, and the Bioethics Committee at the Medical University of Poznań approved the protocol (approval no. 141/18, 2018). The study was completely non-invasive.

### 2.2. Study Procedure

In the first stage of the research, the women participated in nutrition workshops (two meetings, on two consecutive days, completing 1.5 h of work with a dietitian each, in groups of 20 people). During the workshops, under a dietitian’s supervision, the examined women calculated their energy and basic dietary requirements. Additionally, 10-day nutritional plans were prepared based on individual preferences and needs. All the necessary information on the preparation of meals and possible modifications to the diet were also provided. The diet’s basis was to create a 30% energy deficit while maintaining the coverage of the demand for protein, fat, carbohydrates, and minerals and vitamins. The participants were also advised to maintain a daily physical activity of walking at least 5000 to 6000 steps. After the workshops, the examined women were randomly assigned to four groups. The first group (GI) was to use the menu prepared during the workshops and adhere to the physical effort recommendations for the next six weeks. Group II (GII), apart from the diet and recommendations for physical exercise, met a dietitian once a week (50 min) to modify the diet, exchange observations, and control the effects. In Group III (GIII), the participants, instead of meeting with a dietitian, attended adjunctive therapy conducted by a psychotherapist twice a week (2 × 50 min). The participants assigned to the fourth group (GIV) were subjected to both dietician control and supportive psychotherapy.

All the participants were asked to keep a daily physical activity and food consumption diary to control for the effects. Anthropometric parameters were controlled at the beginning of the experiment, after six weeks of therapy, and six months after the end of treatment.

### 2.3. Psychotherapeutic Procedure

The women from GIII and GIV participated in psychotherapy in groups of five people. The psychotherapy took place twice a week in each of the six weeks of the study. The women were offered proprietary supportive therapy in the field of psychodynamic psychotherapy (self-experience). The main goal of the proposed psychotherapy, among other things, was the personal development of the participants, the acceptance of their own bodies, strengthening their egos, and the self-experience of their emotions [31,32]. The same qualified and experienced therapist conducted the psychotherapy for all the participants.

### 2.4. Anthropometric Measurements

All the participants were checked for weight, height, waist and hip circumference, and percentage of body fat at three time points of the study: at the beginning, six weeks after the start of the experiment, and six months following the discontinuation of the procedure. Body weight was measured using a medical scale (with an accuracy of 1 g). Height was determined using a medical altimeter (accuracy up to 1 cm). On this basis, the body mass index (BMI) was calculated. The circumference of the waist and hips was measured with an accuracy of 1 cm using a medical tape measure. The results were used to calculate the WHR (waist–hip ratio). The percentage of adipose tissue in the body was measured using the electrical bioimpedance method using the Tanita BC-545 body composition analyser (with an accuracy of 0.1%).

### 2.5. Statistics

The results are presented as descriptive statistics using the arithmetic mean, standard deviation, range of results, and median. Student’s t-test was used to determine the difference between the means. The chi-square test determined the differences in the distributions of the results concerning the study periods and obesity prevalence. The statistical power of the study with 15 subjects in each group was sufficient to detect, with 80% probability, true differences between groups equal to 2.2 kg/m^2^ for BMI and 0.55 for WHR, under the assumption that the standard deviations of these parameters in the studied population were 2 kg/m^2^ and 0.05, respectively. The calculations were performed using the Excel spreadsheet software from the Office suite and Statistica 13.

## 3. Results

**Figure 1:**
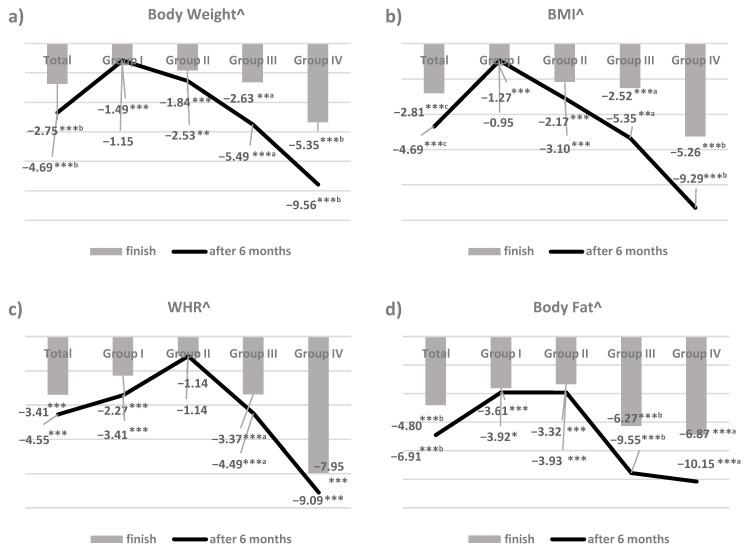
The effect of therapeutic management on selected anthropometric parameters ((**a**) Body Weight; (**b**) BMI; (**c**) WHR; (**d**) Body Fat) of the study women (percentage differences with respect to the baseline results); ^statistical analysis of the differences between the results six weeks (end of the study) and six months after the experiment in relation to the start of the study (at * *p* < 0.05; ** *p* < 0.01; *** *p* < 0.001) and between the end of the research and the period six months after its completion (at a-a *p* < 0.05; b-b *p* < 0.01; c-c *p* < 0.001).

The results obtained in this study are presented in Table 1, Table 2, Table 3 and Table 4 and Figure 1. The participants assigned to the individual groups did not differ significantly in terms of the analysed parameters, measured at the beginning of the study (Table 1). The mean body weight of the studied women was 87.4 ± 0.6 kg (78.0–113.0 kg), while the BMI was 32.0 ± 2.0 kg/m^2^ (28.0–37.7 kg/m^2^). The mean waist and hip circumference and the calculated WHR index did not differ significantly between the groups and amounted to 83.2 ± 6.7 cm, 94.3 ± 6.9 cm, and 0.88 ± 0.05, respectively. Similarly, there were no statistically significant differences between the groups regarding body fat (33.3 ± 2.4%, 29.8–38.8%).

The anthropometric measurements taken after the end of the treatment period are summarized in Table 2. The mean body weight of the study participants was 85.0 ± 5.8 kg (75.0–105.0 kg); the BMI, 31.1 ± 2.1 kg/m^2^ (26.9–38.1 kg/m^2^); the waist circumference, 79.3 ± 6.5 cm (65.0–91.0 cm); the hip circumference, 93.5 ± 6.5 cm (79.0–110.0 cm); the WHR, 0.85 ± 0.05 (0.74–0.97); and the body fat, 31.8 ± 2.5% (27.0–38.3%). A significantly lower mean body weight was demonstrated in the women in GIV (83.2 ± 3.9 kg) compared to GI (86.0 ± 6.2 kg). By contrast, the mean body weight in GII and GIII (approximately 85.4 kg) did not differ significantly compared to the other groups. Such a connection was not observed in BMI, hip circumference, or body fat content. The mean WHR index was significantly lower in Group IV (0.81 ± 0.04) than in the other groups (about 0.86 ± 0.05), which was due to the lower average waist circumference compared to Groups I and II (76.9 ± 5.1 cm vs. approx. 81.0 ± 6.9 cm).

Six months after the end of the study, the participants were re-examined, and the results obtained at this stage are presented in Table 3. After this time, the mean body weight of the subjects was 83.3 ± 6.0 kg; the BMI was 30.5 ± 2.4 kg/m^2^; the WHR, 0.84 ± 0.06; the waist circumference, 78.2 ± 7.6 cm; the hip circumference, 92.9 ± 6.2 cm; and the mean body fat was 31.0 ± 2.7%. There were statistically significant differences between the mean values of the tested parameters obtained in some groups. A significantly lower mean body weight was observed in Group IV (79.5 ± 3.8 kg) compared to Groups I–III (86.3 ± 7.3, 84.8 ± 5.0, and 82.6 ± 4.9 kg, respectively), which did not differ from each other. Significantly lower mean BMIs were demonstrated in Groups III (30.1 ± 1.4 kg/m^2^) and IV (29.3 ± 2.3 kg/m^2^) compared to Groups I (31.3 ± 3.0 kg/m^2^) and II (31.3 ± 1.8 kg/m^2^). There were no differences in mean BMI between Groups I and II or between III and IV. The lowest mean WHR was observed in Group IV (0.80 ± 0.05) compared to the remaining groups (ca. 0.86 ± 0.05), which did not differ from each other. The results for the average waist circumference were similar (73.7 ± 6.6 vs. ca. 80.0 ± 7.0 cm). The mean hip circumference did not differ significantly between the groups. A statistically significantly lower mean content of body fat (ca. 30.2 ± 2.4%) in the women in Groups III and IV was demonstrated compared to Groups I and II (ca. 31.9 ± 2.9%). No statistical differences were observed in the mean body fat between GI and GII or between GIII and GIV.

At the beginning of the study, all the subjects were overweight and obese. Based on the BMIs (> 30 kg/m^2^), there were obese people in Groups I to IV: 85, 87, 87, and 87%, respectively (Table 4). Similarly, the percentages of women with WHR > 0.85 at the beginning of this study were the following: 73, 75, 70, and 72%. In Groups I–III, the body fat was over 30% in 100% of the women, and in Group IV, in 93% of the women. With regard to the BMI, there were changes in the proportions in the distributions of the results. Each of the tested therapy systems was effective in reducing the percentage of obese people in the groups. A statistically significant (χ^2^ = 8.9, *p* < 0.01) effect of the 6-week diet (GI) on the reduction of obesity in the group (from 87 to 67%) was demonstrated. This percentage was also maintained six months after the end of therapy. This change was not statistically significant in GI, considering all three periods (1, 2, and 3). In GII, a similar, statistically significant (χ^2^ = 8.6, *p* < 0.01) change in the percentage of obese people (from 87 to 70%) was recorded. In this group, the analysis, including the examination after six months, showed a further significant (χ^2^ = 12.3, *p* < 0.01) shift in the subjects from obesity to overweight (up to 67% obese). A more pronounced effect of the weight-loss procedure was demonstrated in GIII and GIV. The introduction of a psychotherapy session during the diet (GIII) highly significantly (χ^2^ = 13.3, *p* < 0.001) reduced the percentage of obese people from 87 to 65% in GIII. This effect increased significantly (χ^2^ = 35.9, *p* < 0.001) six months after the end of therapy, reducing the percentage of obese people by another 18%. A more marked, statistically significant effect of diet therapy was demonstrated in the group in which the dietitian and psychotherapist supported the participants during the treatment (GIV). The percentage of obese people in this group decreased from 87 to 53% (χ^2^ = 27.5, *p* < 0.001) and, after another six months, to 40% (χ^2^ = 29.0, *p* < 0.001).

All the applied therapeutic procedures were effective in reducing the WHR indices of the subjects; also, in this case, the combination of a diet, consultations with a dietitian, and psychotherapeutic sessions (GIV) had the most pronounced effect. After the end of the therapy, a statistically significant (χ^2^ = 42.9, *p* < 0.001) shift in the subjects from the obese group (WHR > 0.85) to the overweight group (WHR: 0.80–0.84) was demonstrated, and there were also people showing normal WHR (<0.79); the obese–overweight–normal values changed from 72–28–0% to 30–47–23%, respectively. Six months after the end of the therapy, this effect was significantly amplified (χ^2^ = 68.5, *p* < 0.001), as a result of a further reduction in the percentage of obese people and a further increase in the number of people with normal WHR (20–47–23%, respectively). The impact of the procedures on reducing the number of people with WHR> 0.85 was also shown in the remaining groups, but the effect was not so apparent.

All the adopted weight-loss procedures (GI–IV) resulted in a similarly significant (χ^2^ = 9.9–17.5, *p* < 0.001) reduction in the percentage of women with adipose tissue contents exceeding 30% (from 100% in GI–III and 97% in GIV to 88, 83, 83, and 70% of people, respectively). Six months after the end of the study, the percentages of people with body fat over 30% also statistically decreased (to 80, 78, 73, and 59%, respectively), most significantly in the group where diet therapy was combined with a dietitian consultation and psychotherapy (GIV: χ^2^ = 31.3, *p* < 0.001).

Figure 1 presents the effect of therapeutic management on selected anthropometric parameters of the studied women (percentage differences in relation to the baseline results). Considering all the women, regardless of the group to which they were assigned (total results), the weight-loss procedures applied highly significantly (*p* < 0.001) reduced the average body weight by 2.75%, BMI by 2.81%, WHR by 3.41%, and body fat by 4.80% of the initial parameters. These changes were sustained during the six-month post-study period, and for the mean body weight, BMI, and body fat, there were further reductions from baseline of 4.69, 4.69, and 6.91%, respectively.

When analysing the mean changes in body weight in the individual groups, the highest, highly significant (*p* < 0.001) decrease in this parameter compared to the baseline results was observed in GIV (by 5.35%), and the lowest, in GI (by 1.49%). In GII, there was a reduction in mean body weight by 1.84% (*p* < 0.001) compared to the baseline, and in GIII, by 2.35% (*p* < 0.01). After another examination, six months after the end of the therapy, a statistically significant further decrease in mean body weight in relation to the baseline was observed in GIII by 5.49% and in GIV by 9.56% (difference from the result at the end of the study: *p* < 0.05 for GIII and *p* < 0.01 for GIV). In the case of GII, six months after the end of the study, the mean body weight remained at a reduced level after treatment. Only in GI did the re-measurement of body weight show that the people in this group returned to the baseline result after the period of no treatment; the same trends were observed for BMI.

A statistically significant reduction (at *p* < 0.001) in WHR compared to the baseline value in GIII (by 3.37%) after the treatment was observed. This index significantly (at *p* < 0.05) also decreased after re-examination to the value of 4.49%. In the case of GI and GIV, statistically significant (at *p* < 0.001) decreases in the WHR parameter after treatment (by 2.27 and 7.95%, respectively, compared to the baseline result) were also maintained after the period without treatment. However, there was no statistically significant difference between the result after therapy and six months after its completion. In GII, the mean WHR score did not change significantly, either after the treatment or within six months after its completion.

A statistically significant (at *p* < 0.001) decrease in the mean body fat content of the GI (by 3.61%) and GII (by 3.32%) women was demonstrated. This result did not change six months after the end of therapy. In the case of GIII, a significant (at *p* < 0.001) reduction in the body fat content in the women by 6.27% compared to the baseline results was demonstrated, and a further significant reduction in this parameter was observed in the post-treatment period (by 9.55%; the difference compared to the result after the treatment was significant at *p* < 0.01). Similar effectiveness in reducing mean adipose tissue content was observed in the women from GIV (by 6.87% after treatment and 10.15% after the next six months; the difference between these results was significant at *p* < 0.05).

## 4. Discussion

A lack of a long-term effect in standard obesity treatment attempts suggests that a more complex approach is needed. For this reason, psychotherapeutic approaches such as supportive techniques in the fight against overweight/obesity are of increasing interest [15,18,21,22,24,25,26,27,33,34,35,36,37]. The rationale behind this is that psychological problems often lie at the root of increased body weight [21,26,38]. Apart from satisfying hunger, food today has begun to take on additional roles. For example, it may fulfil an emotional void or distract a person from accumulating problems or a stressful lifestyle, which, in turn, may be a straightforward route to obesity [38]. On the other hand, excessive body weight and the accompanying dissatisfaction with one’s appearance may lead to emotional disturbances or even mood disorders, such as depression, that require a psychotherapist or even a psychiatrist’s intervention and significantly extend the return path to normal weight [26].

Our study attempted to assess the effectiveness of supportive psychotherapy in reducing body weight in young overweight and obese women and the long-term maintenance of the reduced weight. The participants of the experiment were offered psychodynamic psychotherapy. The study shows that the strategy integrating a diet with a dietary consultation and regular psychotherapeutic group sessions proved to be the most effective in reducing body weight and body fat and improving anthropometric indices, such as the BMI and WHR, when compared to the other strategies tested (diet + exercises, diet + exercises + dietetic consultations, and diet + exercises + psychotherapy). Moreover, all of the effects achieved in this case were maintained for another six months after the end of the therapy or even enhanced—as shown by the body weight loss and body fat loss. Although data reporting the efficacy of psychodynamic psychotherapy as a supportive intervention during weight-loss treatment are scarce, a few systematic reviews on the use of various BT techniques in the fight against obesity provide support for the employment of psychotherapeutic approaches in slimming therapy [39,40,41]. Shaw et al. presented a review summarizing the assessment of the efficacy of several behavioural and cognitive–behavioural strategies in weight reduction in overweight/obese adults. The authors highlighted the higher effectiveness of BT’s combination with diet/physical exercises than diet/exercises alone [39].

Similarly, only CBT combined with a fat-restriction diet and physical activity brought statistically significant results regarding weight loss in obese patients suffering from eating disorders in contrast to CBT alone, as reported by Painot et al. [40]. Sbrocco et al. compared the effectiveness of behavioural choice treatment with traditional BT. The latter resulted in greater weight losses in the obese participants of the experiments, although only behavioural choice treatment contributed to further post-experiment weight loss, as proved in the follow-up [41]. Encouraging results for CBT in a combination with mindfulness, an Internet-based intervention, and a diet as an obesity-treatment strategy were shown by Ogata and colleagues. Not only did all the participants succeed in losing weight during the 9-week experiment, but they also achieved further weight reduction in the 18-month follow-up. However, a few limitations in the experimental setup—mainly, the small group of participants (3) and lack of a control group—prevented the authors from drawing unequivocal conclusions from the study [28]. In general, most of the reports presenting the use of combined therapy involving BT, a diet, and/or exercises in obesity treatment show greater weight loss than in the case of any of these strategies being used alone [27].

On the other hand, the results of some studies employing CBT or BT as supportive therapy in the fight against overweight and/or obesity are disappointing. As Cooper et al. demonstrated in their randomized control study, neither CBT nor BT showed long-term efficacy in weight reduction in obese women. Although the experiment participants initially lost weight, most of them regained it, as depicted in the long-term follow-up study [35]. Gade et al., likewise, showed that CBT as a supportive preoperative intervention in bariatric patients was more successful in diminishing mood and anxiety symptoms than in weight reduction, as found one-year post-surgery [42].

The generally inconclusive data regarding the effectiveness of CBT/BT as supportive therapy in obesity treatment may be due to the high heterogeneity of the studies performed. As Lovemann et al. noticed, the problems may include an unstandardized definition of behavioural techniques, varied types of ongoing support (e.g., individual contact or group meetings, telephone calls, and virtual contact), and a wide range of therapists conducting CBT, such as psychologists, weight-loss advisers, and dietitians, to name only a few [43].

There is undoubtedly a need for a permanent change in eating and lifestyle behaviours in overweight/obese patients to achieve satisfying weight reduction and long-term maintenance. However, this may be insufficient in preventing weight regain, as the key problem underlying the obesity may remain unsolved. Traditionally, eating disorders, such as compulsive overeating, night eating syndrome, or emotional eating, have been neglected in standard interventions aiming at weight loss [21]. This approach seems to be evolving as eating disorders are increasingly perceived as originating from early-life experience and treated as addictions [19,21,44]. For this reason, psychodynamic psychotherapy that aims to help patients to cope with deep-rooted psychological problems might be a useful tool in supporting traditional obesity-treatment approaches.

Nevertheless, there are incomparably fewer reports on the use of psychodynamic psychotherapy than on BT in obesity/overweight treatment. Beutel et al. compared psychodynamic psychotherapy’s and BT’s effectiveness in treating severely obese patients (BMI = 36–74 kg/m^2^). They found both therapies to be equally effective in reducing body weight [45]. Similarly, Kiesewetter et al. reported a successful (5%) weight reduction in pre-obese and obese patients undergoing psychodynamic psychotherapy [22]. Our study emphasizes psychodynamic psychotherapy’s high efficacy in combination with diet, physical activity, and dietetic consultations in weight reduction, which significantly contributes to the state of the art in obesity-treatment approaches.

Obesity is a very complex disease with numerous medical and non-medical underlying factors—genetic, endocrine, psychological, social, and environmental, to name a few [46,47]. For this reason, only a comprehensive treatment of this medical condition, integrating traditional approaches (diet and increased physical activity) with psychotherapeutic interventions and pharmacology/bariatric surgery—if recommended—can be successful in the fight against it. Such an integrated approach could become the standard treatment for obesity in the future, primarily if traditional methods, such as diet and increased physical activity, fail. Although our study proves the effectiveness of the combined approach in obesity treatment, a longer follow-up and larger experimental groups are required to confirm its sustained effects. An interesting extension of our research in the future could be the evaluation of the effectiveness of integrated therapy or psychotherapy alone in the treatment of obesity in postmenopausal women, the elderly, or people with obesity-related diabetes.

## 5. Conclusions

Overall, our findings prove the effectiveness of supportive psychotherapy, in the form of psychodynamic psychotherapy, for the treatment of obesity in young women. Although the most significant effects in long-term weight, BMI, WHR, and body fat reduction were demonstrated for a combined approach integrating diet, physical activity, dietetic consultations, and adjunctive psychotherapy, the latter combined with diet and exercise, but without dietary consultation, also produced noticeable results. Additionally, future studies would be required to verify the results collected in other groups of obese/overweight people, for instance, in obese postmenopausal women or patients with obesity-accompanying diseases.

## Figures and Tables

**Table 1:** The anthropometric parameters of subjects at the beginning of the study.

	Total *n* = 60	GI *n* = 15	GII *n* = 15	GIII *n* = 15	GIV *n* = 15
Body weight (kg)	87.4 ± 6.0	87.3 ± 5.9a	87.0 ± 5.3a	87.5 ± 7.5a	87.9 ± 5.1a
	(78.0–113.0)	(78.0–99.0)	(80.0–95.0)	(82.0–113.0)	(80.0–100.0)
	87.0	88.0	85.0	85.0	89.0
BMI (kg/m^2^)	32.0 ± 2.0	31.6 ± 2.4a	32.3 ± 1.8a	31.8 ± 1.9a	32.3 ± 1.9a
	(28.0–37.7)	(28.0–37.7)	(28.7–35.0)	(29.4–36.9)	(28.3–36.9)
	31.6	31.2	32.4	31.2	32.0
Waist (cm)	83.2 ± 6.7	83.5 ± 7.7a	83.2 ± 6.8a	82.4 ± 7.2a	83.5 ± 4.8a
	(69.0–94.0)	(69.0–93.0)	(72.0–94.0)	(69.0–92.0)	(74.0–90.0)
	85.0	87.0	85.0	84.0	85.0
Hip (cm)	94.3 ± 6.9	94.6 ± 5.9a	94.5 ± 6.3a	92.5 ± 5.8a	95.4 ± 8.8a
	(79.0–112.0)	(86.0–104.0)	(84.0–106.0)	(86.0–102.0)	(79.0–112.0)
	94.5	96.0	94.5	89.0	98.0
WHR	0.88 ± 0.05	0.88 ± 0.05a	0.88 ± 0.05a	0.89 ± 0.06a	0.88 ± 0.06a
	(0.80–0.99)	(0.80–0.97)	(0.81–0.97)	(0.80–0.99)	(0.80–0.99)
	0.88	0.89	0.86	0.90	0.88
Body fat (%)	33.3 ± 2.4	33.2 ± 2.4a	33.1 ± 2.0a	33.5 ± 2.4a	33.5 ± 2.9a
	(29.8–38.8)	(30.0–38.8)	(30.5–38.3)	(30.0–38.8)	(29.8–38.8)
	33.4	33.0	33.2	33.6	33.4

Data presented as mean ± standard deviation (SD), (range), and median; a,b—statistically significant differences between groups of subjects at *p* < 0.05. WHR (waist–hip ratio).

**Table 2:** The anthropometric parameters of subjects after six weeks of study (end of therapy).

	Total *n* = 58	GI *n* = 14	GII *n* = 14	GIII *n* = 15	GIV *n* = 15
Body weight (kg)	85.0 ± 5.8	86.0 ± 6.2b	85.4 ± 5.5ab	85.2 ± 6.7ab	83.2 ± 3.9a
	(75.0–105.0)	(75.0–100.0)	(76.0–95.0)	(80.0–105.0)	(76.0–88.0)
	85.0	87.0	84.0	84.0	85.0
BMI (kg/m^2^)	31.1 ± 2.1	31.1 ± 2.5a	31.6 ± 2.0a	31.0 ± 1.5a	30.6 ± 1.9a
	(26.9–38.1)	(27.6–38.1)	(28.3–34.3)	(29.4–34.3)	(26.9–35.3)
	30.8	30.5	32.0	30.5	30.5
Waist (cm)	79.3 ± 6.5	80.3 ± 7.6b	81.5 ± 6.1b	78.5 ± 6.2ab	76.9 ± 5.1a
	(65.0–91.0)	(65.0–91.0)	(72.0–90.0)	(69.0–88.0)	(70.0–85.0)
	80.0	82.0	83.0	80.0	78.0
Hip (cm)	93.5 ± 6.5	93.8 ± 5.7a	93.5 ± 5.2a	91.7 ± 5.3a	95.1 ± 8.7a
	(79.0–110.0)	(85.0–103.0)	(84.0–100.0)	(85.0–102.0)	(79.0–110.0)
	93.5	96.0	94.0	89.0	98.0
WHR	0.85 ± 0.05	0.86 ± 0.05b	0.87 ± 0.05b	0.86 ± 0.05b	0.81 ± 0.04a
	(0.74–0.97)	(0.76–0.93)	(0.78–0.94)	(0.78–0.97)	(0.74–0.89)
	0.85	0.85	0.86	0.86	0.80
Body fat (%)	31.8 ± 2.5	32.0 ± 2.5a	32.0 ± 2.1a	31.4 ± 2.5a	31.2 ± 2.4a
	(27.0–38.3)	(28.7–38.3)	(28.6–37.9)	(28.0–37.0)	(27.0–35.5)
	31.7	31.3	32.0	30.3	30.6

Data presented as mean ± standard deviation (SD), (range), and median; a,b—statistically significant differences between groups of subjects at *p* < 0.05. WHR (waist–hip ratio).

**Table 3:** The anthropometric parameters of the subjects six months after therapy.

KERRYPNX	Total *n* = 51	GI *n* = 12	GII *n* = 12	GIII *n* = 14	GIV *n* = 15
Body weight (kg)	83.3 ± 6.0	86.3 ± 7.3b	84.8 ± 5.0b	82.6 ± 4.9b	79.5 ± 3.8a
	(74.0–99.0)	(74.0–99.0)	(78.0–95.0)	(76.0–93.0)	(74.0–89.0)
	82.5	86.0	84.0	82.0	78.0
BMI (kg/m^2^)	30.5 ± 2.4	31.3 ± 3.0b	31.3 ± 1.8b	30.1 ± 1.4a	29.3 ± 2.3a
	(25.4–37.3)	(27.6–37.3)	(28.4–34.3)	(28.3–34.2)	(25.4–32.3)
	30.3	30.5	31.9	29.7	29.4
Waist (cm)	78.2 ± 7.6	80.3 ± 8.8b	81.1 ± 6.4b	77.7 ± 5.9b	73.7 ± 6.6a
	(60.0–92.0)	(62.0–92.0)	(70.0–90.0)	(69.0–88.0)	(60.0–82.0)
	80.0	81.0	83.0	80.0	74.0
Hip (cm)	92.9 ± 6.2	93.9 ± 6.0a	93.4 ± 5.1a	91.5 ± 5.3a	92.8 ± 7.8a
	(79.0–105.0)	(84.0–105.0)	(84.0–100.0)	(85.0–102.0)	(79.0–105.0)
	93.0	96.0	94.0	89.0	94.0
WHR	0.84 ± 0.06	0.85 ± 0.06b	0.87 ± 0.05b	0.85 ± 0.05b	0.80 ± 0.05a
	(0.69–0.97)	(0.72–0.94)	(0.80–0.94)	(0.78–0.97)	(0.69–0.89)
	0.83	0.85	0.85	0.85	0.80
Body fat (%)	31.0 ± 2.7	31.9 ± 3.0b	31.8 ± 2.3b	30.3 ± 2.3a	30.1 ± 2.6a
	(27.0–38.3)	(28.0–38.3)	(28.0–37.9)	(28.0–37.0)	(27.0–36.5)
	30.9	31.3	32.0	30.0	29.3

Data presented as mean ± standard deviation (SD), (range), and median; a,b—statistically significant differences between groups of subjects at *p* < 0.05. WHR (waist–hip ratio).

**Table 4:** Percentage distributions of the results for time points of the study and analysed parameters.

Group	GI			GII			GIII			GIV		
Time point	1	2	3	1	2	3	1	2	3	1	2	3
BMI > 30.0	85	67	67	87	70	67	87	65	47	87	53	40
BMI 25.0–24.9	15	33	33	13	30	33	13	35	53	13	47	60
χ^2^ (*p*)	8.9 (0.003)			8.6 (0.003)			13.3 (0.000)			27.5 (0.000)		
	5.5 (0.064)			12.3 (0.002)			35.9 (0.000)			29.0 (0.000)		
WHR > 0.85	73	60	53	75	73	65	70	60	53	72	30	20
WHR 0.8–0.84	27	33	40	25	20	35	30	33	40	28	47	47
WHR < 0.79	0	7	7	0	7	0	0	7	7	0	23	33
χ^2^ (*p*)	6.6 (0.037)			4.9 (0.087)			5.5 (0.063)			42.9 (0.000)		
	11.1 (0.025)			12.8 (0.012)			11.1 (0.025)			68.5 (0.000)		
Body fat > 30%	100	88	80	100	83	78	100	83	73	93	70	59
Body fat < 30%	0	12	20	0	17	22	0	17	27	7	30	41
χ^2^ (*p*)	9.9 (0.002)			15.6 (0.000)			15.6 (0.000)			17.5 (0.000)		
	18.6 (0.000)			20.8 (0.000)			27.0 (0.000)			31.3 (0.000)		

Time points: 1—beginning; 2—after six weeks of therapy; 3—six months after therapy. WHR (waist–hip ratio).

## Data Availability

The data that support the findings of this study are available on request from the corresponding author (R.W.W.). The data are not publicly available due to their containing information that could compromise the privacy of the research participants.
